# Astrocytic ion channel Kir4.1 deficit underlies chronic pain

**DOI:** 10.4103/NRR.NRR-D-25-00773

**Published:** 2025-09-29

**Authors:** Sarah Mountadem, Daniel L. Voisin, Radhouane Dallel

**Affiliations:** Université Clermont Auvergne, CHU Clermont-Ferrand, Inserm, Neuro-Dol, Clermont-Ferrand, France; Université de Bordeaux, Inserm, Neurocentre Magendie, Bordeaux, France

While acute nociceptive pain is a crucial warning system that protects us from injury or disease, chronic pain is not protective, but a pathological condition. As such, it is now recognized as a disease in its own right, which major classes refer to inflammatory, neuropathic, and idiopathic pain. It is frequent, with up to a third of the population that may suffer at one point from chronic pain. It is often associated with other pathologies, including sleep disorders, anxiety, depression, and is still difficult to treat. It thus represents a significant burden in terms of health and societal impact (Tracey et al., 2019). The mechanisms of chronic pain involve multiple diverse pathways in both the peripheral and central nervous systems (CNS), reflecting its multifaceted biology. Indeed, research over the past decades has established that central sensitization (enhancement in the function of neurons and circuits in central nociceptive pathways), in particular within the dorsal horn, the first central relay of nociceptive inputs plays a key role in the chronicity of pain (Latremoliere and Woolf, 2009).

A wealth of evidence highlights the critical role of central astrocytes and their associated chemical mediators to maintain chronic pain as well as central sensitization (Ji et al., 2019). Astrocytes react to stimuli or conditions that induce pain by significant morphological and molecular changes, in parallel with alterations of physiological properties, and such changes are closely correlated with behavioral manifestations of pain hypersensitivity (Ji et al., 2019). Consequently, astrocytes are crucial promoters of pain pathogenesis through crosstalk with neurons and microglia. However, the cellular mechanisms underlying changes in astrocyte properties are still unknown. The inward rectifier potassium channels Kir4.1 might be key actors in these processes. They are highly expressed by astrocytes, making their predominant background K^+^ conductance. They are critically involved in extracellular K^+^ homeostasis, maintenance of the astrocyte resting membrane potential, facilitation of glutamate uptake and astrocyte cell volume regulation (Ohno et al., 2021). As a consequence, alterations in astrocyte Kir4.1 channel function or expression could disrupt astrocyte cell volume regulation, extracellular K^+^ buffering, and glutamate clearance, thereby profoundly influencing neuronal excitability. Accordingly, reduced Kir4.1 channels function or expression has been associated with various CNS pathologies such as depression, epilepsy, Huntington’s disease, Alzheimer’s disease, amyotrophic lateral sclerosis, autism spectrum disorders, and spinal cerebellar ataxia (Ohno et al., 2021). More recently, loss-of-function of central Kir4.1 was shown to underlie a novel form of glia-neuron interaction in the dorsal horn that causally leads to the development of chronic pain (Zhang et al., 2022; Ou et al., 2023; Mountadem et al., 2025). We put in perspective these findings, since they uncover Kir4.1 as a putative target to treat chronic pain.

**Figure 1:**
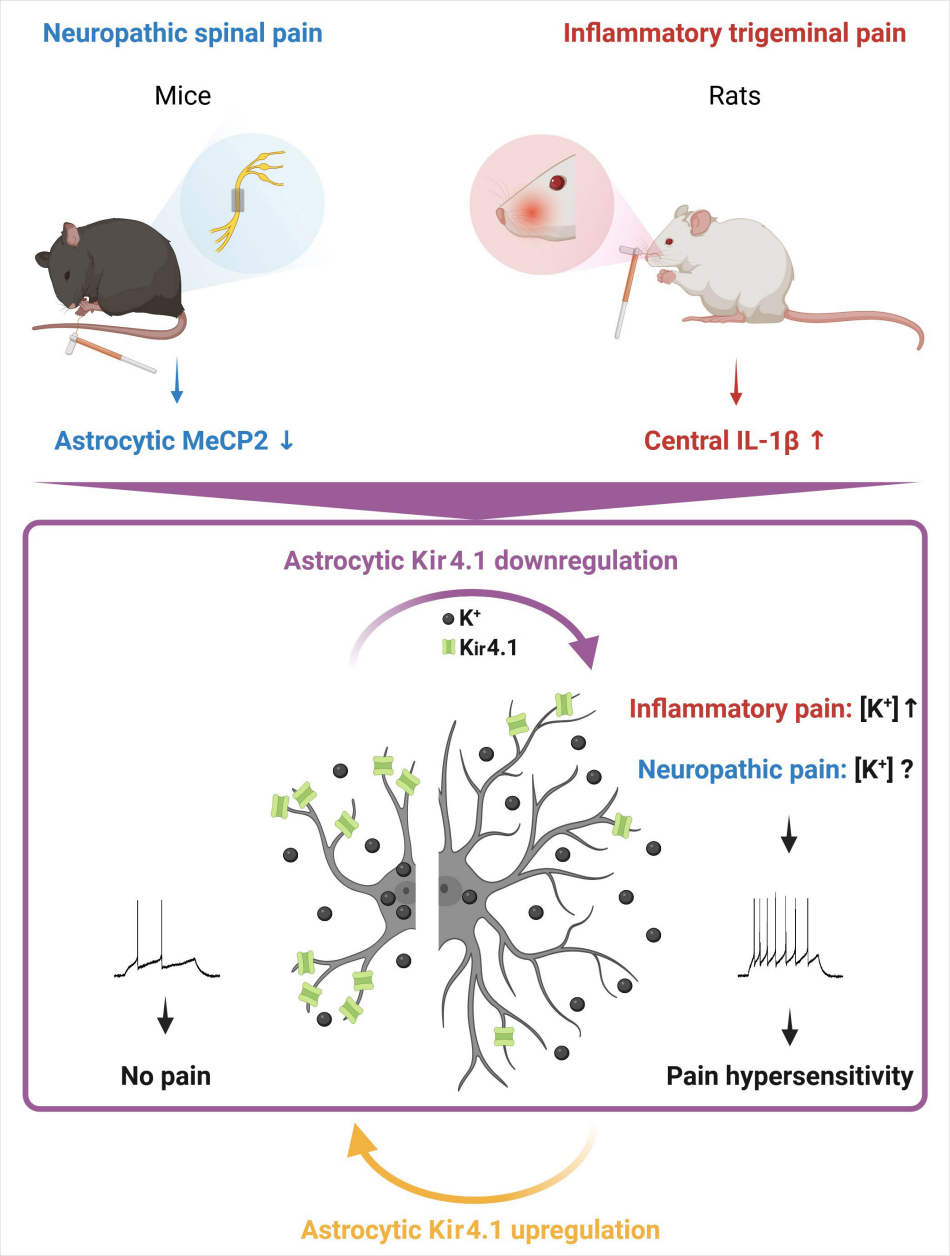
Astrocytic ion channel Kir4.1 deficit underlies neuropathic spinal pain and inflammatory trigeminal pain. Chronic constriction injury of the mouse sciatic nerve induced Methyl-CpG-binding protein 2 (MeCP2) downregulation in spinal cord astrocytes (top left) and persistent facial inflammation in male rats induced an up-regulation of interleukin-1β (IL-1β) in the medullary dorsal horn (top right). In both cases, the result was a downregulation of dorsal horn astrocyte Kir4.1 channels that drove hyper-excitability of dorsal horn neurons and pain hypersensitivity. Astrocyte Kir4.1 downregulation drove inflammatory pain hypersensitivity through altered [K^+^] baseline levels. Restoring Kir4.1 function prevented pain hypersensitivity induced by nerve lesion and inflammation. Created with BioRender.com.

Zhang et al. (2022) first reported that Kir4.1 expression was reduced in the rat medullary dorsal horn (MDH), the first central trigeminal pain relay, after the inferior alveolar nerve was transected. Ou et al. (2023) investigated whether reduced expression of Kir4.1 channels in astrocytes contributes to pain symptoms in a mouse model of chronic neuropathic spinal pain (**Figure 1**). They first found that the expression levels of Kir4.1 were decreased in spinal astrocytes after chronic constriction injury (CCI) of the sciatic nerve in both males and females as shown by reverse transcription polymerase chain reaction and western blot analyses. Further single-cell RNA sequencing analysis showed that expression of both Kir4.1 and Methyl-CpG-binding protein 2 (MeCP2) was diminished in astrocytes following CCI. MeCP2 is a transcription factor that regulates the expression of the gene encoding Kir4.1. General conditional knockdown of the Kir4.1 channel, as well as specific knockdown in spinal astrocytes induced hyperalgesia in mice. When Kir4.1 channels were overexpressed in spinal cord hyperalgesia behaviors were relieved in both Kir4.1 conditional knockout mice and CCI mice. Electrophysiological recording in spinal slices from Kir4.1-siRNA mice showed Kir4.1 channel function was diminished in astrocytes, and firing patterns of neurons in the spinal dorsal horn were altered, consistent with impaired neuronal responses to spontaneous conditions and/or moderate stimulation. Finally, astrocyte-specific knockdown of MeCP2 induced hyperalgesia in mice, while overexpression of MeCP2 in astrocytes increased Kir4.1 expression and reduced hyperalgesia in the CCI mouse model. Altogether, these findings highlight the critical role of spinal Kir4.1 reduction in driving hyperalgesia in chronic neuropathic pain, under the regulation of astrocytic MeCP2.

Using a rat model of persistent inflammatory trigeminal pain, Mountadem et al. (2025) found that the sustained pain hypersensitivity following injection of the pro-inflammatory compound complete Freund’s adjuvant (CFA) into the face of animals was associated with increased neuronal activity within the MDH, which was paralleled with local astrocyte activation (**Figure 1**). At the same time, MDH astrocytes displayed striking changes in morphology and membrane properties, including depolarization of their resting membrane potential, and increases in their membrane resistance and capacitance. These changes were in line with the specific downregulation of Kir4.1 channel function and expression in MDH astrocytes that followed CFA injection and were a probable consequence of the central release of interleukin-1β during the inflammatory process. Importantly, when Kir4.1 function was reduced in naive rats by means of the delivery of a dominant-negative form of Kir4.1 to MDH astrocytes, this was sufficient to induce trigeminal pain hypersensitivity and hyper-excitability of superficial MDH neurons. Conversely, when a selective gain-of-function in Kir4.1 was virally produced in MDH astrocytes, this prevented both pain hypersensitivity and hyper-excitability of superficial MDH neurons in the inflammatory pain model. Since DNA methylation regulates Kir4.1 transcription, it was also possible to restore astroglial Kir4.1 expression using a DNA methyltransferase inhibitor, 5-azacytidine, which systemic administration reduced pain hypersensitivity induced by CFA. These two last sets of experiments established that the downregulation of astrocyte Kir4.1 in the MDH is required to produce pain hypersensitivity. Furthermore, Mountadem et al. (2025) demonstrated that the increased K^+^ baseline levels that were observed in the MDH after persistent inflammation resulted from the downregulation of Kir4.1 and were an essential mechanism leading to hyperexcitability of superficial MDH neurons and central sensitization. While the contribution of astrocytes to pain hypersensitivity has been attributed mainly to their release of signaling molecules, this work provided the first direct evidence that dorsal horn neuron hyper-excitability may mainly result from an interruption of the normal capacity of astrocytes to maintain potassium homeostasis. On top of that, trigeminal pain hypersensitivity and the downregulation of MDH astrocyte Kir4.1 after CFA were sex-dependent, since they developed in male rats only. This difference was related to the fact that female rats developed a much less severe local inflammation at the CFA-injected facial site, suggesting that female rats display mechanisms that protect them against the inflammatory effect of CFA, which would reduce input to the CNS. This was site specific, as inflammation and pain were the same in females and males following CFA injection in the paw. Altogether, these findings establish that MDH astrocyte Kir4.1 downregulation is causally involved in driving inflammatory persistent pain hypersensitivity in male rats. Of note, Kir4.1 channels expressed by satellite glial cells in the trigeminal ganglia have been involved in both inflammatory and neuropathic trigeminal chronic pain (Lin et al., 2025). Although these important glial cells do share some functional similarities with astrocytes, they differ in their location, as they remain in the peripheral nervous system, in the active molecules they release, the neuronal components they interact with, and when they are activated during chronic pain states.

The results from these studies strengthen the growing evidence implicating central Kir4.1 channels in the pathophysiology of various pathological conditions of the CNS, leading to neurological and psychiatric diseases (Ohno et al., 2021). Mountadem et al. (2025) ascertain a convincing mechanism that drastically affects neuronal activity when the downregulation of a single ion channel induces astrocyte dysfunction. It is also possible that increases in extracellular K^+^ alter synaptic transmission, and thus contribute further to neuronal hyper-excitability. Such dysregulation of extracellular K^+^ has been involved in the pathophysiology of various neurodegenerative diseases and has recently been shown to distinguish healthy ageing from neurodegeneration (Ding et al., 2024). Further work using other models on brain pathologies is needed to establish whether it is a common factor of several pathological conditions of the CNS.

More specifically, the converging results from these studies highlight the role of astrocytic Kir4.1 ion channel deficit as a crucial mechanism of chronic pain (**Figure 1**). In the chronic constriction injury of the mouse sciatic nerve, the downregulation of spinal dorsal horn astrocyte Kir4.1 channels resulted from MeCP2 downregulation in spinal cord astrocytes and drove hyper-excitability of spinal dorsal horn neurons and somatic pain hypersensitivity. In the persistent facial inflammation in male rats, the downregulation of trigeminal dorsal horn astrocyte Kir4.1 channels likely resulted from central interleukin-1β upregulation and drove hyper-excitability of trigeminal dorsal horn neurons and facial pain hypersensitivity. In both models, restoring Kir4.1 function prevented pain hypersensitivity. A key question moving forward is whether astrocyte-specific loss-of-function of Kir4.1 also occurs in inflammatory pain models, implicating the somatic sensory system, and in trigeminal neuropathic pain models, as well as in other mouse and rat models of chronic pain, for instance, cancer-related. That would define astrocyte-specific loss-of-function of Kir4.1 as a common central mechanism of chronic pain. There is also a need to further explore the sex-specificity of alteration in Kir4.1 function in all models and understand the underlying mechanisms. Two different pathways leading to astrocytic Kir4.1 down-regulation were identified. While Ou et al. (2023) found in the CCI model that spinal Kir4.1 expression was regulated by MeCP2, Mountadem et al. (2025) suggested that inflammation-induced MDH astrocyte Kir4.1 downregulation was dependent upon central interleukin-1β. Whether different or similar regulatory mechanisms are involved in different chronic pain models, as well as the involvement of other cellular actors, such as microglia, will need to be investigated. Mountadem et al. (2025) showed that Kir4.1 drove alterations of neuronal activity and facial inflammatory pain hypersensitivity through altered K^+^ clearance. Whether this is also the case in other chronic pain models will have to be checked. Beyond reduced K^+^ buffering, astrocytes could promote pain through impaired glutamate clearance, altered brain-derived neurotrophic factor expression, and the release of several signaling molecules, such as gliotransmitters and chemokines. In a near future, a systematic analysis of dorsal horn astrocyte gene expression based on transcriptomics should highlight potential unexpected mechanisms resulting from Kir4.1 downregulation. A remaining important question will be to determine whether Kir4.1 ion channel deficit is also a feature of chronic pain in humans. If so, the results obtained in these studies suggest that targeting central Kir4.1 could be of therapeutic potential to treat chronic pain. This could be achieved using drugs recently identified to enhance Kir4.1 currents, notably, the pro-resolving lipid mediator maresin 1. Maresin 1 is a ligand of GPR37L1, an astrocytic orphan G protein–coupled receptor that has been recently associated with chronic pain in preclinical models (Xu et al., 2025).

*This work was supported by funding from Institut National de la Santé et de la Recherche Médicale (Inserm), Université Clermont Auvergne (France), CHU Clermont-Ferrand (to RD), the French government IDEX-ISITE initiative 16-IDEX-0001 (to RD), and The Fondation pour la Recherche Médicale (FRM) (to SM).*
